# Supplementary figures and images for: Evaluation of the performance of the Influenza-like Illness (ILI) surveillance system in the Okai Koi North District, Greater Accra Region, 2022
Source: PLoS One. 2025 Sep 19;20(9):e0332334. doi: 10.1371/journal.pone.0332334 (PMC12448339; doi:10.1371/journal.pone.0332334)

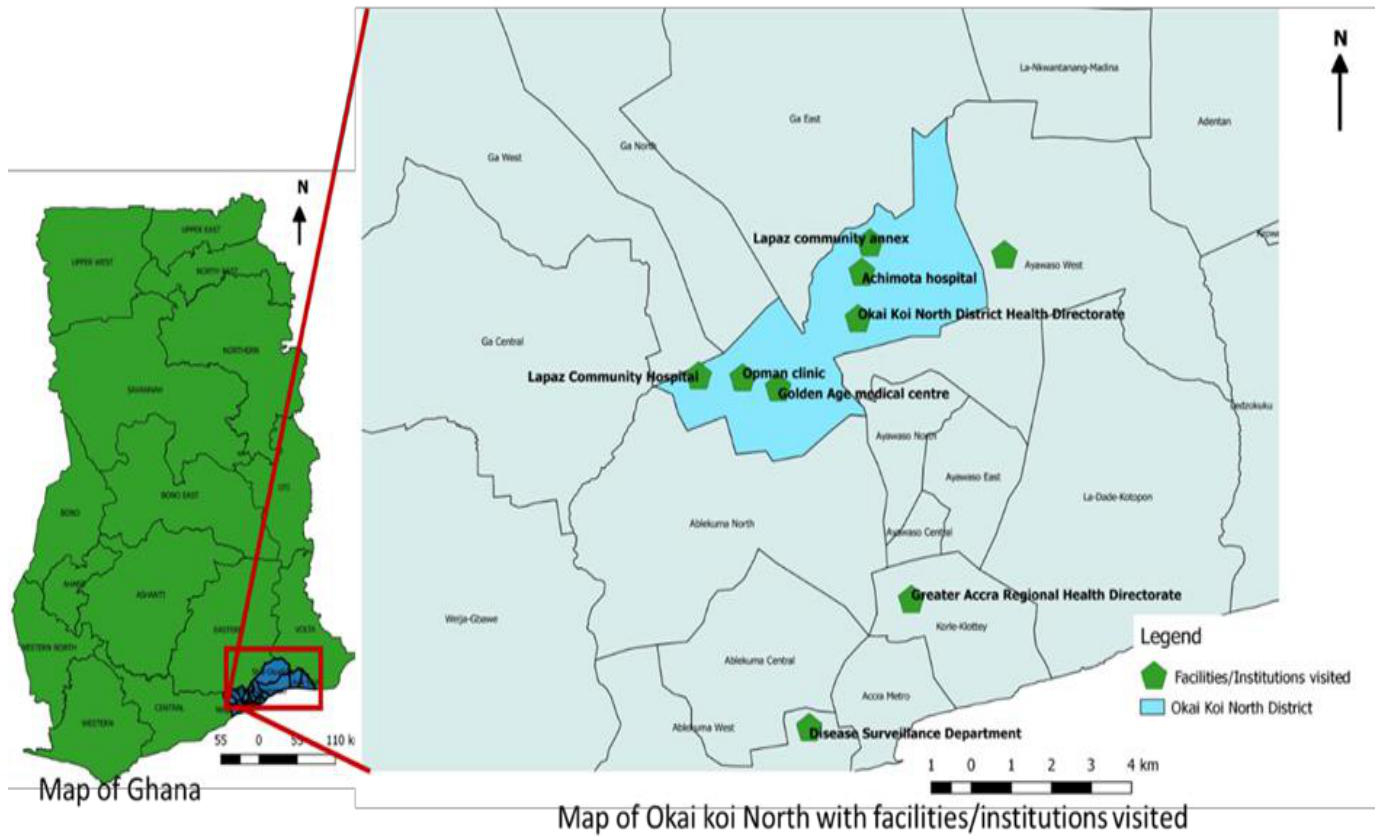

Supplement: S1 Fig — (TIF) [file pone.0332334.s001.tif]

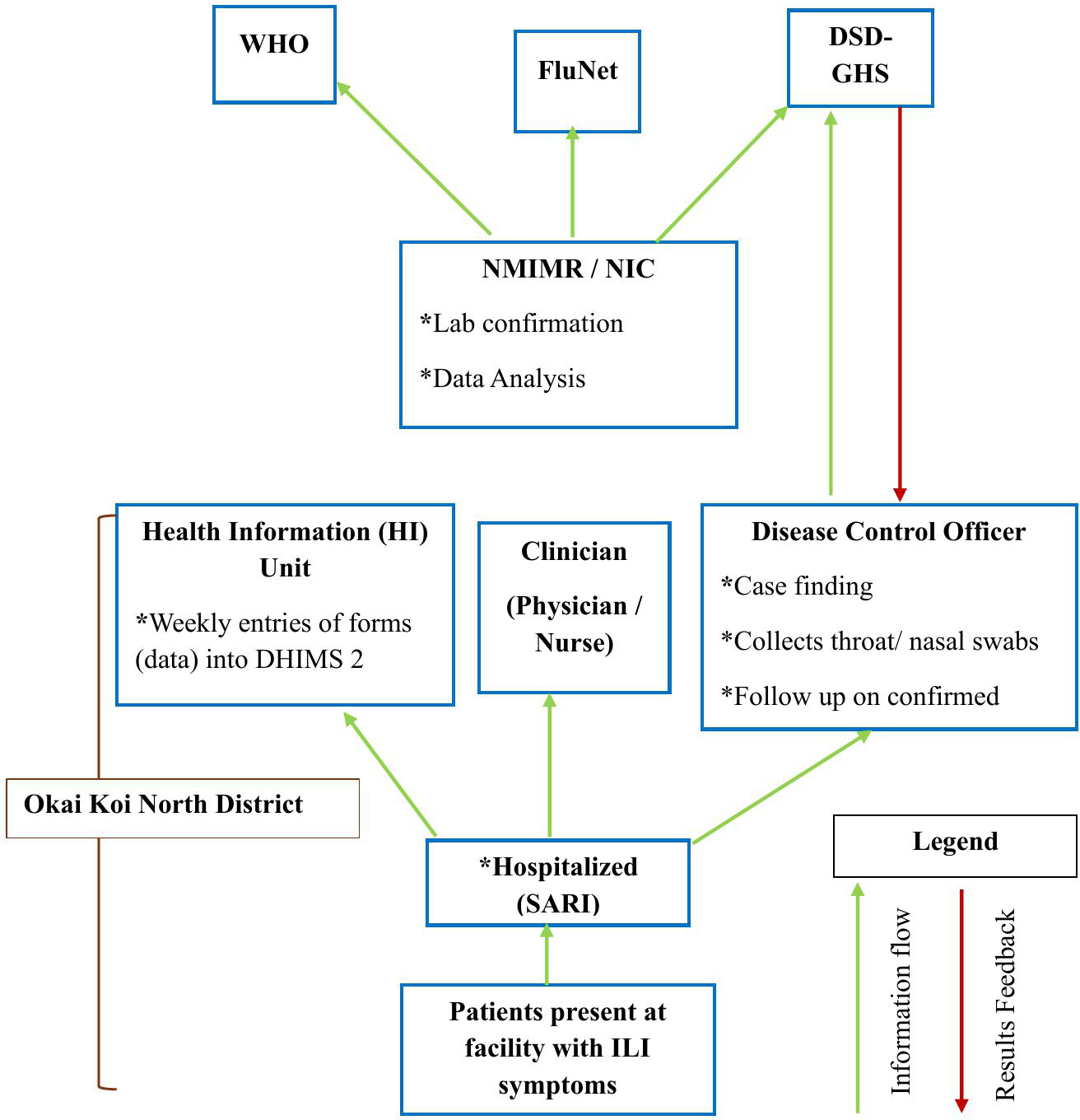

Supplement: S2 Fig — (TIF) [file pone.0332334.s002.tif]

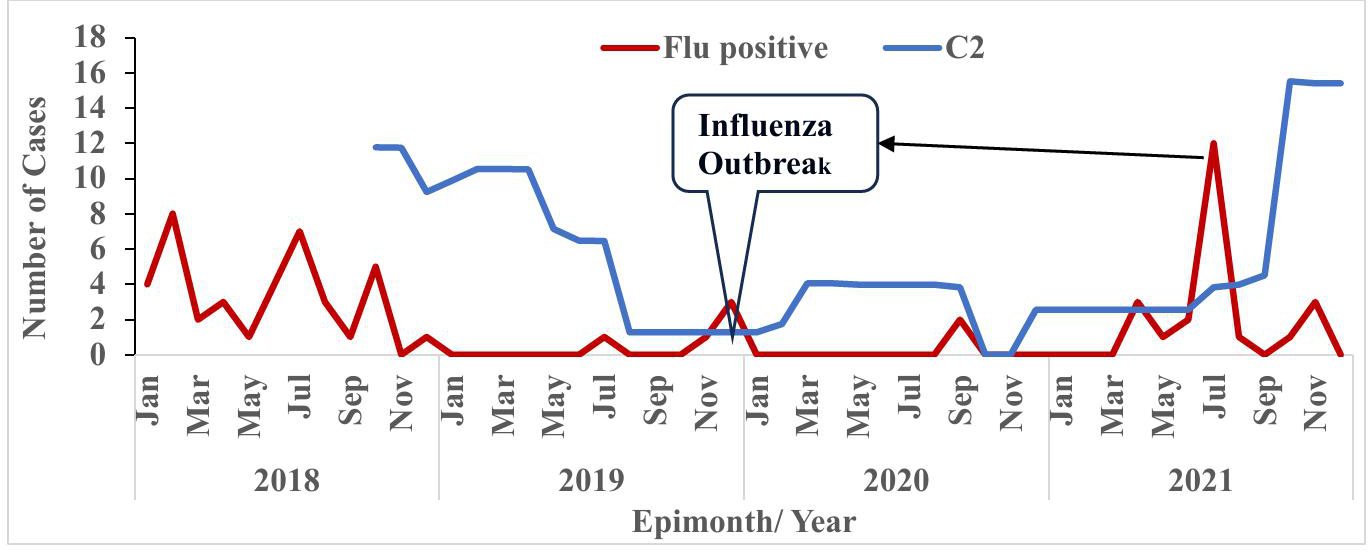

Supplement: S3 Fig — (TIF) [file pone.0332334.s003.tif]

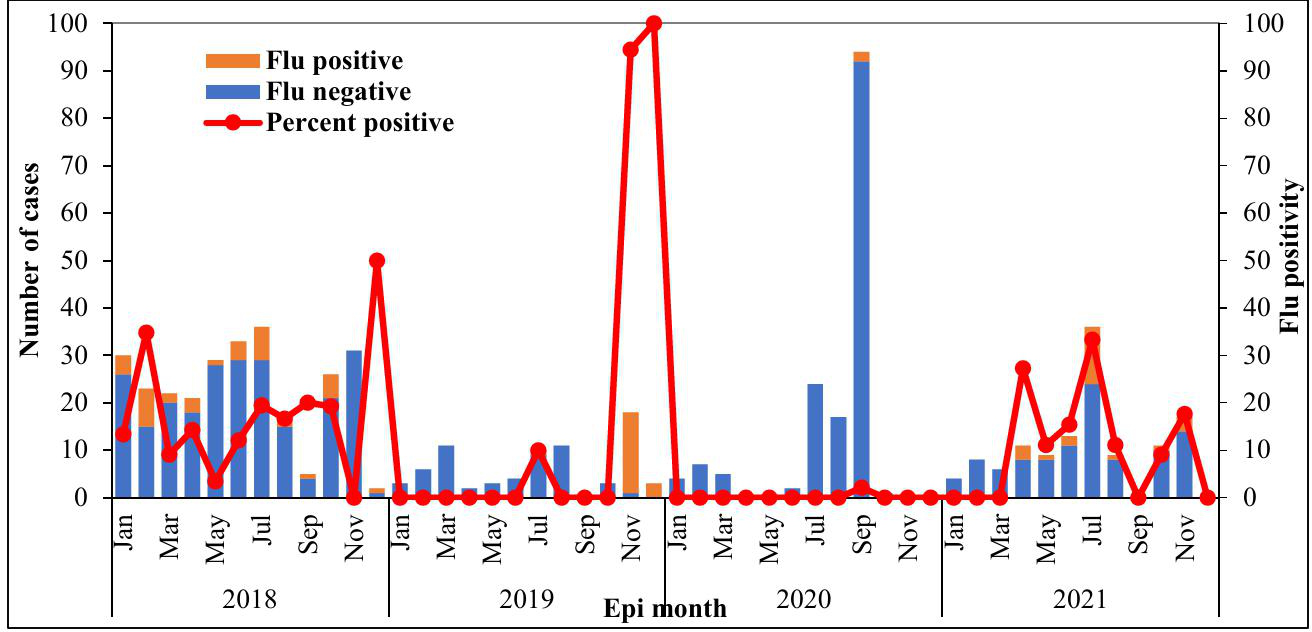

Supplement: S4 Fig — (TIF) [file pone.0332334.s004.tif]
