# Supplementary material for: Evaluation of the performance of the Influenza-like Illness (ILI) surveillance system in the Okai Koi North District, Greater Accra Region, 2022
Source: PLoS One. 2025 Sep 19;20(9):e0332334. doi: 10.1371/journal.pone.0332334 (PMC12448339; doi:10.1371/journal.pone.0332334)
Supplement: S5 File — (DOCX) [file pone.0332334.s005.docx]

1. **QUESTIONNAIRE FOR STAKEHOLDERS**

**Assessment of the performance of Influenza-like Illness surveillance system in Okai Koi North District, Greater Accra Region, 2022**

**Questionnaire for stakeholders at the health directorate/ NMIMR/NIC/ national level and health facilities**

This questionnaire is strictly for academic purposes. No respondent or the organization he/she represents will be identified by name in the report.

Your input to the following questions will help improve the Influenza-like illness surveillance system in the Okai Koi North District.

Unique identifier for respondents **…………………………………….**

Date of interview **…………………………………….**

**A. Demographic Characteristics**:

| A1. Level of Surveillance Participation A2. Name of facility/ institution:  Health Facility  Sub-District A3. Number of Health Staff involved in surveillance:  District ___________________________________________ | |
| --- | --- |
| A4. Age (years):  …………………… | A5. Sex:  Male Female |
| A6. Category of Health Worker/ Occupation A7. Position: | |
| A8. Your main activity in the ILI surveillance System: | |
| A8. Department: | A9. Number of years worked: |
| A10. Received Training on IDSR/ ILI:  Yes No: | A11. If yes, Year of Training: |

**B. Purpose and Operations of the system**

1. Do you know the objectives of the ILI Surveillance? Y/N

State any if YES……………………………………………………………………

2. What is the planned and actual use of the data generated?...........................................

3.What is the case definition for ILI surveillance?...........................................................

**Bi.**  **Case Definition:**

| Code | Assessment (Assign one point for a correct answer and zero for a wrong answer for each item mentioned) |  |
| --- | --- | --- |
| Bi1. | Fever > 38 ºC |  |
| Bi2. | Onset of symptoms and difficulty in breathing within the last 10 days |  |
| Bi3. | Cough |  |
| Bi4. | Sore throat |  |
| Bi5. | Severe ILI symptoms and requires hospitalization (SARI) |  |
| Bi6. | Person of any age in which clinician suspects ILI |  |
|  | **Total Score** |  |

4. What is the location of the surveillance system?..................................................

**SAMPLE COLLECTION, STORAGE AND TRANSPORTATION**

1. Do you collect ILI samples at this level (Routine / Outbreaks)? Y/N
2. Do you send / receive ILI samples/ data each week? Y/N

If ‘No’ why not? ................................................................................................................

1. How many ILI patients do you sample/ expect in a week? = Number of SARI patients? =
2. ALL patients who meet the ILI/ SARI case definition
3. First five patients who meet the ILI/ SARI case definition
4. First ten patients who meet the ILI/ SARI case definition
5. None
6. What kind of respiratory specimen do you collect from patients who meet the ILI/SARI case definition
7. Nasopharyngeal specimen and/or Oropharyngeal specimen
8. Nasal aspirate c. Mouth Swab d. None of the above
9. Does your facility have the following?
10. ILI sampling algorithm? Y/N
11. SOPs? Y/N
12. Who collects the sample for laboratory testing?.............................................................
13. Is the person responsible for sample collection trained? Y/N
14. How long does it take you to complete a case-based form? (Mins) ……………………………
15. Where do you store the respiratory specimen temporarily before sending it to NMIMR
16. Fridge 2-8°C b. Freezer -20 to-80°C c. On the bench d. None of the above
17. How long do you store the specimen before sending to NMIMR
18. Within 48hrs b. Above 48hrs c. One week d. One month e. Other……….
19. How do you package the sample for shipping?
20. Single packaging b. Double packaging c. Triple packaging
21. Is the sample transported under cold chain (4-8°C)? Y/N And within 48hours after collection? Y/N

**COMPONENTS OF SYSTEM**

1. What is the catchment population for the ILI surveillance?............................................
2. Who enters the data……………………………………………………….
3. How is data stored? Is it under lock and key (password)?..............................................
4. Do you keep copies of the case-based forms (both new and filled out ones)?
5. Yes b. No
6. Do you analyze the influenza surveillance data generated from your facility (Y/N) and by who?.......................................................................

If YES, how……………………………………………………..

1. Do you chart both ILI/SARI suspected cases and confirmed cases in your facility?
2. Yes b. No
3. Do you review the distribution of influenza cases by person, place and time?
4. Yes b. No

If YES, how often…………………………………………………

1. Do you report ILI/SARI cases on the IDSR (Y/N) and to what level?..............................
2. Do you receive the results for samples you send to NMIMR? Y/N

If YES, how often ………………………………………………………….

1. How do you rate sending of samples to NMIMR?
2. Difficult 🞏 b. Somewhat difficult 🞏 c. Somewhat easy 🞏 d. Easy 🞏

10b. If the response is “difficult” or “somewhat difficult,” please explain why

…………………………………………………………………………………………….

1. What is the flow chart of information flow?..................................................................

**SYSTEM REQUIREMENT**

1. Is there budgetary allocation for ILI surveillance system? (Y/N); Mention the sources
2. What percentages of your time is spent on ILI surveillance and follow up?
3. What other resources do you need for ILI surveillance reporting?

a. Training?

b. Supplies?

c. Travel?

d. Computer and other equipment?

e. Other related services?

1. Can you describe any instances when ILI surveillance data led to changes in policies or programs at your level?

**In General, What Are Your Overall Impressions of the Influenza Sentinel Surveillance System?**

**……………………………………………………………………………………………………………………………………………………………………………………………………………..**

**Do You Have Any Suggestions for Improving the Influenza Surveillance Network’s Operation?**

**………………………………………………………………………………………………………………………………………………………………………………………………………………**

***Thank you***

1. **CHECKLIST ON ILI SYSTEM ATTRIBUTES**

| **Attributes** | **Items** | **Yes (Y)/ No(N)** |
| --- | --- | --- |
| Usefulness | 1. Is the ILI Surveillance System meeting its objectives? 2. Estimates the magnitude of morbidity and mortality 3. Detection of disease in a timely manner 4. Identify trends in the disease occurrence (using thresholds) 5. Detects epidemics 6. System stimulates research intended to lead to prevention and control 7. Are the data generated from the ILI surveillance used for any public health action? 8. Other anticipated uses of data generated ………………………………………………….. |  |
| Simplicity | 1. Is the case-based form easy to fill? 2. Simple flow of information i.e. CHPs/Health facility to sub-district to district to region to national   (Number of reporting levels……………………)   1. Is the case definition simple and standard? 2. Amount of follow up period and updating of cases? STATE period…………………………….. 3. Data analysis methods simple at your level? 4. Laboratory requirement (complexity) |  |
| Flexibility | 1. It accommodates/ respond to other event/new demand e.g., COVID-19, H3N2 2. Staffs are skilled, they easily adapt/accommodate changes in case definition. 3. Variation or change in focal person (personnel) affects system’s performance |  |
| Data  Quality | 1. Completeness and clarity of the case reporting form (< 90%)   *Randomly sample 30 case-report forms*   1. How many cases of samples were reported in the last 1 year (verify with reports) 2. Availability of mechanisms for monitoring/controlling any errors 3. Data values recorded in the DHIMS2 are consistent with the registers/records at the reporting source |  |
| Acceptability | 1. Full participation of local, national, and multinational agencies 2. Completeness of reporting forms (< 90%) 3. Is the facility/ level meeting the expected samples per week or consistently collect Flu samples within the past 3 or 6 months? 4. Timeliness of data reporting from lower level (e.g., CHPS/ Health facilities) 5. Feedback availability, from higher levels (e.g., national) to the lower level |  |
| Sensitivity | 1. Has the system detected any epidemics (outbreaks) in the past 5 years in your catchment area?  **qualitative** 2. Percentage/ proportion of ILI cases detected by surveillance system (Compare with lab diagnosis data) **quantitative**   *Calculate Sensitivity of over 5 years* |  |
| Predictive value positive (PVP) | 1. Proportion (%) of suspected cases **confirmed** by laboratory testing   *Calculate PVP of 5years (data from Nationals) * |  |
| Representativeness | 1. Does the ILI surveillance data accurately represent people residing in Okai Koi District? 2. Characteristics of population under surveillance e.g., age, sex, socioeconomic status, occupation, geographic location (distribution of cases) 3. Are samples coming from both out-patients and in-patients? |  |
| Timeliness | 1. Average time between symptom onset and reporting to hospital 2. Average time between collection of samples and submission to laboratory for testing 3. Average time between collection of samples by laboratory and provision of test result to reporting facility 4. How often do they receive feedback from samples or cases sent to NMIMR or testing labs? | ……………….  ……………….  ……………….  ………………. |
| Stability | 1. Availability of regular funding for system operation? 2. Did your site experience a shortage/rupture in the following supplies over the past 6 months? 3. Case-based forms: Never 🞏 Once 🞏 2-3 times 🞏 More than 4 times 🞏 4. Influenza sampling kit (swabs, VTM, etc): Never 🞏 Once 🞏 2-3 times 🞏 More than 4 times 🞏 5. Number of times internet connection was down for more than 24 hours within the past 1 month: Never 🞏 Once 🞏 2-3 times 🞏 More than 4 times 🞏 6. Number of times electricity cut (including generator)? :   Never 🞏 Once per week 🞏 2-3 times per week 🞏 More than 4 times per week 🞏   1. Does the system operate during shortages and rupture of the above listed in 2? 2. Is there back-up in place in terms of power outages and system computer breakdown? 3. Proportion of time system is operating fully (Are staff always available to run the ILI surveillance within the facility’s operational time? 4. Is the data easily accessible for use by those who generate the data and stakeholders? |  |
