# Supplementary material for: Evaluation of the performance of the Influenza-like Illness (ILI) surveillance system in the Okai Koi North District, Greater Accra Region, 2022
Source: PLoS One. 2025 Sep 19;20(9):e0332334. doi: 10.1371/journal.pone.0332334 (PMC12448339; doi:10.1371/journal.pone.0332334)
Supplement: Attachment — Submitted filename: Response to Reviewers.docx [file pone.0332334.s007.docx]

**Rebuttal letter addressing each point raised by academic editors and reviewers**

**Major review points:**

1. In the introduction, provide a crisper statement of the study’s main objectives in the final paragraph to guide readers toward the methods. And consider emphasizing the importance of thresholds in the context of outbreak detection slightly earlier, so that readers understand the significance of that gap when it appears later in the paper.

Response: The study’s main objective has been made more crisper in the final paragraph of the introduction, found on lines 66-68 of the introduction. It reads as “We present here findings on our evaluation of the ILI surveillance system in Okai Koi North District in assessing the systems performance in concert with its usefulness and system attributes”.

The importance of threshold has also been included earlier in the introduction, in the context of outbreak detection. This can also be found on line 49-56 of the introduction and it reads as, “These Flu outbreaks usually meet the lower levels of surveillance, example the district levels unprepared to mitigate them which either goes undetected or delayed detection. The Flu situation is not the same for all diseases, as some diseases under surveillance here in Ghana, example Measles have epidemic thresholds set for them. Thresholds denote a degree of incidence that is essential for timely detection of disease outbreaks, and its response”.

2. Strengthen the explanation of how thresholds were retroactively generated using CUSUM.

Response: The generation of thresholds for the district using CUSUM-C2 method has been explained into details at the data analysis section under materials and methods. It can be found on line 174-182 to read as follows;

“We generated an ILI epidemic threshold for Okai Koi District from data garnered from the NIC using the CUSUM - C2 method. The four years data were arranged by year and months (January – December). For each threshold point, we took the mean and standard deviation for a group of seven surveillance points (months over the four years) in the past, skipping over the two most recent points (months). We then added three times the standard deviation to the mean value to obtain the C2 value for specific point/month [C2 = Mean + 3(standard deviation)]. The calculated C2 values for the months were used to plot the epidemic threshold chart using Microsoft Excel”.

3. Provide further explanation on the mismatch between sentinel site numbers and NIC records, including potential reasons (e.g., lost samples, incomplete form submission).

Response: This has been further explained and reasons allocated for it under the discussion section, on line 524-528. And it reads as follows,

“The reported data discrepancy between the sentinel site and the NIC by our study could be due to non-existing routine/periodic data harmonization, samples received at the NIC without the case investigation forms, and insufficient sample volume due to leakage. This discrepancy could also be as a result of the surge and prioritization of COVID-19 cases in 2021, where some ILI cases from the district could have been treated as COVID-19 cases”.

4. Provide a short guidance or example for how local health facilities could routinely apply thresholds (C-2, CUSUM, etc.) to perform outbreak detection, since a minimum background of disease surveillance and data analysis would be required for that, what implies in granting local capacity and workforce towards this training. Also, how early warning system approach could be incorporated in this.

Response: This has been addressed under conclusion, line 601-620, to read as,

“At the district and health facility levels health information (HI) unit responsible for data analysis and management could be rigorously trained by the data managers at the NIC or national level on the use of Microsoft Excel to generate and chart the ILI and other diseases’ threshold alerts. Epidemiological threshold levels at the healthy facility levels should be generated by epidemiological weeks to support the early detection of surge in ILI cases, which will prompt heightened surveillance through active case search. Ghana health service has data platforms for every surveillance level, such as DHIMS-2 and SORMAS (surveillance, Outbreak Response Management and Analysis System), with enhanced text messaging features incorporated in them, the HI teams could receive and share prompt messages when an alert/epidemic threshold is met”.

5. Addressing in a more detailed way despite the limited generalizability due to single-district focus, how these findings could be instrumental for improving ILI surveillance in similar settings.

Response: This has been addressed under the discussion section, line 568-573. It then reads as “Despite the focus on Okai Koi North District, findings from this study could largely help improve existing ILI surveillance systems since most of the districts in Ghana has similar characteristics especially within the urban regions. The findings from this study could also serve as a baseline data to structure new ILI surveillance systems in other districts of Ghana and sub-Saharan Africa”.

6. Since potential recall or reporting biases are possible in self-reported data about timeliness and system operations were possible, what be the directions to overcome this during building author's conclusions?

**Response:** We were cognizant about potential recall or reporting bias during stakeholders or surveillance staff interview hence to minimize or reduce the effect of recall bias in our study, we undertook the activities stated below. This can be found under the evaluation approach and data collection technique under the materials and methods section, on line 151-158 and it reads as follows,

“Surveillance data from the NIC, which is data from the sentinel site and DHIMS 2 were reviewed and extracted using the developed checklist. Also, we reviewed consulting room registers and OPD records from all the health facilities visited within the evaluation period, using the developed checklist. The evaluation targeted information from records (data and forms) on all in-patients and out-patients, males and females reporting at these visited health facilities in the district as well as data on samples received at the NIC. Similarly, surveillance activities were also observed using the checklists. The purpose of the observation carried out related stakeholders’ answers to questionnaire to what was practiced on the field”.

7. An addition of future perspectives for ILI surveillance could be helpful for readers understanding towards the evolving process of this area. The following reference is just one of examples on how ILI surveillance have been developed in new aspects: <https://publichealth.jmir.org/2023/1/e46644/>

Response: This has been addressed under the discussion section, on line 574-589, to read as,

“Globally, disease surveillance is taking a new turn with new approaches and technologies such as participatory surveillance and web-based surveillance could improve participation in the traditional ILI surveillance in Ghana. Participatory surveillance (PS) is an evolving strategy which when incorporated, actively engages community members and support community volunteers to improve reporting on health incidents on a regular basis to obtain data to monitor trends. In the broader perspective, PS may reveal indications of an early outbreak by showing signs of an emerging disease or a concentration of symptoms in particular locations especially hard to reach communities. On the other hand, web-based surveillance is another direction of public health surveillance system that leverages on web-applications platform such as, social media, twitter, to track disease patterns with the Ghanaian population using aggregate data from search queries.”

Minor review points:

8. Watch for typographical inconsistencies (e.g., “Okai Koi North” vs. “Okaikoi North”)

Response: This has been resolved in manuscript. Okai Koi North District has been used to replace all typographical inconsistencies regarding its name.

9. Ensure all acronyms (GISRS, NIC, DHD, etc.) are spelled out at first mention.

Response: All acronyms in manuscript have all been spelt out at first mention.
