# Supplementary material for: Evaluation of the performance of the Influenza-like Illness (ILI) surveillance system in the Okai Koi North District, Greater Accra Region, 2022
Source: PLoS One. 2025 Sep 19;20(9):e0332334. doi: 10.1371/journal.pone.0332334 (PMC12448339; doi:10.1371/journal.pone.0332334)
Supplement: Attachment — Submitted filename: Response_to_Reviewers_auresp_2.docx [file pone.0332334.s008.docx]

**Rebuttal letter addressing each point raised by academic editors and reviewers**

**Major review points:**
1. First, the authors have clarified the importance of timeliness by incorporating average time intervals. This level of detail had been highlighted as essential, and now the paper supplies numerical data and acknowledges the proportion of samples that met the time standard. The discussion explicitly interprets how delayed care-seeking contributes to lower positivity rates, which aligns with the earlier suggestion to connect patient behavior to detection performance. Although they do not present exact turnaround times for lab feedback beyond stating an average two days once reagents are available, they mention weekly result transmissions and specify the 58% compliance rate for 48-hour sample delivery. That partially satisfies earlier comments.

Response: This section has been revised or improved regarding turnaround time (TAT) for specimen testing as seen under the discussion section, on line 488 - 493.

“Although our findings indicate an average of 48hours for specimen testing, weekly submission of results and reports to sentinel sites and global platform, system does not capture data on date of specimen testing at the NIC which refutes the calculation of turn-around-time of specimen testing which could impact result feedback for patient care. Data reconciliation as proposed earlier using the SORMAS platform could remedy this gap, where specific date of testing of patients’ specimen could be completed”.

2. Threshold development was flagged as a gap before, and the authors now include an account of how they retroactively generated a C-2 threshold for the four-year dataset. This addition underscores that December 2019, April 2021, and July 2021 would have been epidemic signals. They explicitly recognize the absence of a real-time threshold in the district and recommend establishing one. That is a significant improvement over the previous version because it engages with the notion of an “early warning system” more clearly. The paper nevertheless does not describe a formal plan or next steps for implementing thresholds, though it does reference training local staff on using software like Microsoft Excel to plot alerts. This suggests a partial but not comprehensive adoption of the recommendation to institutionalize such a threshold.

Response: A more comprehensive plan to institutionalize thresholds have been included in discussion section, on line 355 – 362 and to read as;

“A national implementation of threshold development for the ILI surveillance system could be achieved through stakeholder engagement, to review the national influenza protocol for the inclusion of a mandatory development of thresholds, at national, regional, district and sub-district levels. Hence include national level training of all surveillance staff at the lower levels on thresholds development, data entries for its development and interpretations of thresholds. Also, SORMAS (surveillance, Outbreak Response Management and Analysis System), a data platform used by GHS could have its dashboard updated for the calculation of thresholds”.

3. Regarding data harmonization, the authors now make a point of explaining discrepancies in the number of suspected cases recorded at different levels. They attribute the mismatch to limited data reconciliation, missing forms at the NIC, insufficient sample volumes, and especially the shift in priority testing during the peak of COVID-19. This acknowledgment indicates they have attempted to address the data harmonization issue, though it remains more of a descriptive explanation rather than a structured solution. The recommendation for routine data reconciliation is briefly included, but it is not expounded upon in detail.

Response: A detailed expounded data reconciliation has been provided also under discussion section, on line 420 – 444, and this reads as;

“Efforts aimed at curbing data discrepancies in the ILI system could be through an implemented and routine stakeholder quarterly meeting, between both national (GHS and NIC) and district (sentinel site). These routine meetings could be held at the sentinel site where missing information including patients’ outcome, are reconciled, using the source data (i.e., patients’ folders). It also offers an opportunity for the facility to update their forms or data routinely with the results from the NIC lab.

More robustly, the adaptation of SORMAS at the national level which was used during the COVID-19 era could be tweaked to include the capture of influenza data entry interface at all levels as well as at the NIC. The adaptation of SORMAS will foster the mandatory/required completion of all essential data variable on the questionnaire. Through SORMAS, the NIC would be able to update the specimen test results field in real-time for use at the district or health facility level to aid patient care.

To resolve the issue of insufficient sample volumes, the NIC and stakeholders at the national level should periodically train lower-level staff and research assistants that transport samples, on specimen handling. As the NIC continues to supply the Flu site with VTM (viral transport medium), they need to train both their research assistants and the staffs at the sentinel site on the proper cocking of VTMs and thus, safe handling during sample collection and transportation. Whereas COVID-19 samples were prioritized during the pandemic, we will recommend that during the event of outbreaks and pandemics, laboratory testing which are associated with pathogens of the same family could be conducted concurrently”.

4. On the topic of tracking patient outcomes and mortality, the authors do confirm that none of the case-based forms contain final outcome data, and that mortality is not captured. They cite the need for these fields to be made mandatory but do not detail a formal plan for implementing and monitoring such changes. They do, however, articulate how the lack of outcome data constrains the understanding of disease severity and overall burden, something that the previous review had highlighted as missing. This partial improvement demonstrates awareness of the issue, but further specification on how to close the gap would strengthen this section.

Response: This section has been revised and can be found under the discussion section, on line 365 – 371. This reads as follows;

“The ILI surveillance system could improve or integrate data entry into SORMAS at the facility level, which captures the epidemiological data of the patient and thus, be linked to the data entry platform at the NIC for monitoring and subsequent completion of all the required fields after the results are confirmed. Both sides complete all required fields including disease outcomes which foster proper monitoring. This plan implemented by the stakeholders at the national level will help understand disease severity such as mortality, recovery, lost to follow-up, etc.

5. Finally, the paper addresses sustainability and the risk of reliance on donor funds by noting that staff salaries are covered by government sources but that most lab testing supplies and logistical support come from external partners like WHO or the US CDC. This passage shows that the authors have recognized the over-dependency on external donors, which was a previous concern. However, much like in other areas, the revised text identifies the problem but does not systematically propose a sustainability plan. They do suggest greater national budgeting but only briefly. Therefore, they acknowledge the concern more prominently than before, though they stop short of providing a complete blueprint.

Response: This section has been improved with a blueprint to stop or reduce over-dependency on external donors, and can be found under the discussion section, on line 465 – 476. It reads as;

“In as much as the NIC and the influenza surveillance system in Ghana, has over the years depended on funds from external donors, such as the US CDC, WHO and NAMRU-3 long-terms sustainability of the system remains grey. To resolve the overdependency on external donors, sustainability efforts could be through advocacy to highlight the importance of the ILI surveillance system to the government, for the consideration of budget allocation to support equipment procurement/service contracts, reagents and consumables all year round.

During the COVID-19 era, the NIC experienced a novel and heightened private-public partnership including financial institutions, private institutions contributing towards the procurement of equipment, donation of reagents and consumable. The ILI surveillance system could leverage on this cemented private-public partnership, to advocate for financial support as well as grants towards equipment procurement and service contracts, reagents, and consumables for infection-prevention-control (IPC)”.
